# Educational crowdsourcing to support the learning of computer programming

**DOI:** 10.1186/s41039-015-0011-3

**Published:** 2015-07-21

**Authors:** Dhiya Al-Jumeily, Abir Hussain, Mohammed Alghamdi, Chelsea Dobbins, Jan Lunn

**Affiliations:** Applied Computing Research Group, Liverpool John Moores University, Liverpool, UK

**Keywords:** Open learning, Crowdsourcing, Adaptive learning systems

## Abstract

Recent advancements in technology have enabled a shift to occur in teaching and learning. We are living in a connected world where physical boundaries of attending an institution to gain an education no longer apply. There are currently thousands of courses available online that do not require formal attendance. As such, this era of “open learning” pioneers an innovative research, across multiple disciplines. One domain of knowledge where open learning can be advantageous is within computer science. This industry is now highly in demand and can benefit from open learning platforms, where students may not have the opportunity to formally attend courses but still want to enhance their skills. However, there are some significant limitations in open learning applications: how they assess the quality of learning (i.e., was it just copying or at best *learning by rote*) or considering individual differences among learners. In this emerging research paper, we posit the idea of an adaptive, crowdsourced, and primarily educational technology, targeted at software development students. The proposed technology caters for either individual or group learning. It differentiates itself from other tutoring and programming support technologies as it will continually monitor and assess students’ performance in each phase of the education process.

## Introduction

Teaching novice programmers the skills associated with software development is a challenging process (Kim and Lerch 1997). This is due to the fact that teachers are required to individually assess their students and then, according to their existing level of knowledge and preferred learning styles, start teaching them a number of tasks such as the technical aspects of programming and new ways of thinking to solve problems. Moreover, programming is essentially a technically rooted and practical set of skills. Therefore, beginner programmers need to build their skills in entering code, building software, and then as necessary executing, debugging, and correcting the software. In practical, lab-based sessions, this often needs one-on-one help from teaching staff. With large class sizes and demands on tutoring staff, weak students in particular may not have the opportunity to get the individual help they require (Wang et al. 2011). Furthermore, this becomes increasingly difficult and challenging in an open learning environment where thousands of students can enroll simultaneously and where teachers and students are not physically in the same space.

At the present time, there are no intelligent adaptive or individualized tutoring technologies that satisfactorily solve those above-mentioned issues. Given the online nature of open learning, there is a clear benefit to automated software that can assist in actively tutoring of software development students. Therefore, in this emerging body of research work, we posit a solution of supporting some of the identified limitations in open learning environments. One of our aims of this exploration is to integrate the concept of “assessment for learning” into a learning technology to better fit to student learning capabilities. Furthermore, recognizing and reacting to learners’ preferred delivery styles to improve student performance and increase their engagements into learning materials is another aim of this study. Additionally, we provide an in-depth analysis of some of the issues of crowdsourced educational applications. This is an emerging approach to open learning and open access to education that is useful to explore as information can be gathered from a number of sources. For example, a popular web community, Stack Overflow, is cited as an example of crowdsourced education. It provides a fast “first answer” response time of on average, 11 min, with contributing users rewarded for their participation with a reputation points scheme. Stack Overflow is used by the software development community to share and solve common problems and solutions/suggestions. Its reward scheme encourages contributions while allowing information recipients to judge the perceived quality of the help they are receiving. Educational crowdsourcing applications of this nature support lecturers, students, and professionals in communicating with each other, primarily asking questions and receiving solutions. However, there are still some significant limitations in those applications: how they assess the quality of the learning (i.e., was it just copying or at best learning by rote) or considering individual differences among learners (Mamykina et al. 2011).

This paper discusses an emerging area of research that posits the idea of developing an adaptive, crowdsourced, and primarily educational technology, targeted at software development students. The proposed system will guide students in their learning through interactive feedback and adaptive curriculum delivery that suits both their current level of learning and preferred learning styles. The remainder of this paper is organized as follows. Section 2 briefly describes the background and provides a general overview of the different learning styles of students. This is important consideration in an open learning environment where thousands of students with different learning styles enroll in open learning courses. Therefore, in order for these courses to be truly open, it is important to understand and cater for a wide array of individuals. Section 3 explores the new opportunities that crowdsourcing offers in open education as well as resulting adequate applications. Section 4 details the design of the proposed system, while Section 5 provides a summary of the paper and the future direction of the research.

## Background

Rutherfoord and Rutherfoord (2008) defined learning styles as the characteristic techniques in which learners learn, understand, and acquire information. Some researchers defined a learning style as an approach of learning a concept. This is because each learner has a different preferred approach to understanding or learning subjects. For example, some learners prefer to and perform better when learning visually, while others may prefer to learn aurally (Hawk and Shah 2007; Rutherfoord and Rutherfoord 2008). Considering learning styles of all students in the traditional classroom can be a challenging issue for teachers. Teachers have only a limited time in preparing their materials and delivering their classes, lectures, and tutorials (Watson et al. 2010). Established pedagogical theory specifies several learning style models (Graf et al. 2007), including Kolb Experiential Learning Theory (Hawk and Shah 2007) and the VARK model (Leite et al. 2009). Moreover, each of these models has different descriptions for the learning style. This paper centers on the VARK model as it represents the most widely influential model (Eltigani et al. 2011). Equally, the research approach will also be evaluated for students who learn programming primarily by doing, referred to as kinaesthetic learners, and the VARK model provides the most efficient support for this style. The following subsection discusses the VARK model in more detail.

### The VARK model

The acronym VARK stands for Visual (V), Aural (A), Read/Write (R), and Kinaesthetic (K). Learning style has been defined in this model as a learner’s preferred way of remembering, understanding, and reasoning about knowledge. The VARK model has been used for advising teachers to know the preferred learning styles of their students (Eltigani et al. 2011). Significantly, this model has a supporting validated questionnaire (and website version available at http://vark-learn.com/) that allows a reasonable quick (self) assessment of a learning style preference. This assessment can be done by completing the online questionnaire, which then advises the learners about their learning style based on the VARK learning model supported by the weighted outcomes of the questionnaires. The VARK model defines four learning styles (Hawk and Shah 2007; Eltigani et al. 2011) as follows:

1. *Visual*: one of the original basic learning styles. In this particular type, a learner learns best by seeing. For example, flowcharts, diagrams, maps, and so on.
2. *Aural*: another significant learning style in traditional classroom education. Here, a learner prefers to learn best through listening to lectures, discussion, tapes, etc.
3. *Read*/*Write*: These learners prefer self-directed learning—e.g., reading textbooks, reports, or webpages, and then summarizing or writing what they have understood.
4. *Kinaesthetic*: This is another primary learning style in the classroom. Kinaesthetic learners learn most effectively through experience, undertake experiments, and carry out case studies, practical sessions, etc.

The next section addresses the main characteristics and findings related to established software applications widely recognized as learning style sensitive.

### Learning styles in adaptive systems

There are several adaptive learning systems that, as part of their adaptation process, consider the learning styles of the learners. However, they evidently show limitations (Eltigani et al. 2011) that we explore in this subsection with regard to personalizing the learning process, and compare the various adaptive learning systems with focus on programming skills as follows.

#### iWeaver

This is an adaptive tutoring system used to teach the Java programming language. Wolf (2002) reported that the aim of developing iWeaver was to accommodate individual learning styles in an adaptive e-learning environment. The learning process inside this system is described in the following steps. First, when a learner logins into the system, the system will request from this learner to answer 118 questions of the Building Excellence Survey. Once the survey is completed, the learner is given an explanation of his/her suitable learning style within some recommendations on the media representation for the first content module. Subsequently, the learner is able to study the first module in his/her preferred learning style or other styles. Once the learner finishes studying, the system provides an automated feedback. However, such system is missing some of the important aspects of the teaching and learning processes, for example, there is no pre-assessment to judge on the programming level of the learner. Watson et al. (2010) note that the iWeaver system fails to express any pedagogical meaning beyond a very simplistic representation of the relationships between curriculum elements.

#### Protus

Protus is an adaptive, intelligent web-based programming tutoring system that is also used for teaching the Java programming language. Learner profiles are created with some basic information; then, the learner’s preferred learning style is ascertained via a set of questions. This information is stored in the profile and used to select the appropriate lesson customization for the specific learning style (Klašnja-Milićević et al. 2011). However, this system does not provide any significant functionality with regard to adapting a curriculum towards the learner ability; there is no assessment-driven learning, nor any initial diagnostic assessment. In order to create a truly adaptive system, the learner’s current and developing ability must be tested.

#### AEHS-LS

AEHS-LS, or Adaptive E-learning System based on Learning Styles, is used for teaching the Javascript scripting language. Eltigani et al. (2011) state that it was designed to assess the consequences of adapting educational materials individualized to the student’s learning style. As with the above-explained Protus system, learners create an associated profile during the registration process. Again, the learners are responsible for selecting their appropriate learning style. AEHS-LS prompts the users to select their own learning style, if known, and if not, prompts them with the Fleming VARK questionnaire (Leite et al. 2009). Once the learning style is either determined or selected, lessons are delivered according to the selected style. AEHS-LS defines a strict outcome-concept structure, such that lessons follow a traditional structure outlining concepts, delivering materials, and then summarizing with a plenary. Appropriate style-specific resources are generated for each concept by a subject expert and then simply selected by the software at delivery time. Responses to plenary quizzes are used to monitor performance against a particular style, continually adapting the selected learning style. The AEHS-LS-related publications do not exactly make clear how the learning style adaptation assesses performance against alternative styles.

The analysis of the resulting system showed that AEHS-LS-engaged students outperformed the control group students. However, student feedback demonstrated that the auditory learners experienced difficulty, though this is not attributed to the system’s approach. It is supposed that this is due to audio delivery in a language other than the participants’ native language (Eltigani et al. 2011). The AEHS-LS study does not investigate this further.

The research work in developing adaptive systems has clearly conducted valuable investigations into harnessing technology as a mechanism for adapting curriculum as well as delivery in accordance with a learner’s preferred style. Equally, the studies appear to demonstrate, in limited evaluations, that correctly exploiting a learning style does improve the assessment performance. However, it is evident that the systems do not fully address both the pedagogical and technological concerns regarding learning style-adaptive learning support systems.

Summarizing some of the major missing pedagogical impacts around the above-discussed applications iWeaver, Protus, and AEHS-LS, they have not considered what learners need to be taught as there is no diagnostic assessment embedded. Another shortcoming refers to the fact that the differences among learners have not been thought over in the technologies applied. Against this background, the following section further investigates the interaction between learning styles and technology to provide better clarification of these important aspects of open access to education.

## Methods

This section discusses the interactions between technology and learning styles that constitute of open education resources where the contents as well as the information are distributed and shared between learners who can in turn enhance their knowledge and collaboratively shape materials and experience using technology. With regard to this emerging development, e-learning enables the adoption of various advances of information and communication technology and related applications to support an open education environment. The first subsection looks at how pedagogical research and practice in learning style mapping and application can be exploited in existing technological approaches. The second subsection examines the potential for technology to augment existing pedagogical practice and maximize impact. The final subsection discusses criticism of learning styles—both in the classroom and in the e-learning environments in the context of open learning approaches and the integration of electronic-selected learning styles into teaching.

### The impact of learning styles on technology

Learning styles have several potential areas of impact in existing technology. One such impact is utilization of data about learning styles to improve the quality of e-learning systems’ adaptation models. Intelligent e-learning systems should ideally track the learner’s progress and optimize the learning process to take advantage of the learners’ strengths and help them overcome their weaknesses. There is evidence from recent studies that students who engage with a system that incorporates a learning style track-and-response mechanism outperform those who study outside the system. For example, 70 % of students who used the Protus system to learn the Java programming language found this adaptive system successfully guiding them through the appropriate materials with useful explanations (Klašnja-Milićević et al. 2011).

Another potential impact of including learning styles in learning software is the personalization of the learning experience—and importantly, the increasing engagement. Several of the educational technology systems were designed to suit a variety of learning styles for learners (Klašnja-Milićević et al. 2011; Wolf 2002; Wolf 2007). The vast majority of students engaged with these systems found e-learning systems more enjoyable than the traditional learning systems in the classroom. One significant advantage in this regard is that a well-designed software system can make these identifications and selections with little computation cost, contrasting with the teachers’ effort to correctly identify and respond to all of the learners and their differing learning styles in a large classroom (Watson et al. 2010).

### The impact of technology on learning styles

Just as good pedagogical practice can feed into the design of tomorrow’s open learning systems, technology can continue to feed back into teaching practice. For example, lecturers already engage their students more thoroughly through the use of additional multimedia content (Stickel 2009). Additionally, technology provides a means to reach a wider range of students (Wolf 2002; Singh and Holt 2013). However, there is a significant advantage of open learning—in terms of the potential for increasing teaching and learning output, letting subject experts focus on material creation, and automating much of the repetitive tasks. Deferring time-consuming tasks to a software system allows greater one-on-one teaching and learning time, a challenging prospect in the traditional classroom (Wolf 2002; Singh and Holt 2013).

Technology can facilitate the rapid assessment of many learners’ learning styles using an open learning approach. For example, iWeaver determines the learning style of their users by asking them over 100 multiple choice questions, with the system automatically providing the content in their preferred learning styles.

However, technological tools do not yet suit all of the types of learning styles. This is due to the fact that teaching materials are not always adaptable to all types of learning styles. Generally, some subject matters and topics do not lend themselves to all the VARK styles. Equally, certain kinaesthetic learning tasks, especially devoted to gaining, for example, better programming style and software development skill, are recently still ill suited to an electronic or virtual environment. For example, the Tablet PC is a teaching tool used in engineering courses. Kinaesthetic learners evaluated this tool as un-engaging, while visual learners found it an enjoyable classroom addition, and they have a greater preference for it (Stickel 2009).

### Learning style criticism

By looking at the above-explained studies on introducing learning styles in adaptive e-learning systems, there is a big debate. Eltigani et al. (2011) found that including learning styles in an e-learning system helped to improve students’ achievement and performance. Conversely, Brown et al. (2007) reported that there is no evidence to support the idea that matching learning styles to learners improves learning effectiveness, although their sample was primary school children. Popescu (2009) criticized the learning style approach for several reasons. One objection is that there is a large number of learning style models, with no unanimously accepted approach. Additionally, the length of the assessment questionnaires was considered to discourage participants. Popescu (2009) suggested that learning style questionnaires should be revised for use in web-based learning systems as they ignore technology-related preferences.

Additionally, the authors of this paper have identified further issues in integrating electronic-selected learning styles into teaching. One significant issue is that of the teaching workload, particularly for those tutors tasked with creating their own materials. Designing several sets of much the same material, each tuned to a particular learning style is likely to be very time-consuming, requiring a considerable increase in effort.

Another issue refers to some subjects that are naturally not suitable to be taught in accordance with a particular style. For example, teaching heavily verbose mathematics or programming subjects would be very difficult to engage the auditory learners. Also, developing materials for auditory learners may create other challenges as the student’s language may differ from the delivery language. Eltigani et al. (2011) noted that auditory learners who natively speak Arabic found that listening to spoken English by a non-native is difficult. Therefore, those above-discussed issues should be taken into a consideration by a near-future study. More importantly, in this paper, we aim not only at discussing the explained issues in treating learning styles but also at considering them and tackling as many of them as possible in our envisaged automated system. To counterfeit the limitations largely identified by now, we will apply a trend-setting interdisciplinary approach, namely merge adaptive systems with open learning solutions with special reference to crowdsourcing support as detailed in the next section.

## Crowdsourcing in open education

Related to the rapidly broadening development of open learning and open access to education, the role of the individual in a social context increases giving humans the particular chance to collectively shape various dimensions of life. The crowdsourcing was recently the answer to this development providing enormous chances for designing different application areas in a novel manner, such as education, financing, and entrepreneurship. There are a wide range of definitions that can be applied to the term “crowdsourcing.” One of those definitions is that crowdsourcing can be an online community facilitating a large group of people from across the globe to meet with each other in order to discuss and exchange ideas, solve problems, and share entertainment (Brabham 2008). However, Estellés-Arolas and González-Ladrón-de-Guevara (2012) refine and integrate this definition of crowdsourcing to say that it is a “participative online activity” including a group of community stakeholders of “varying knowledge, heterogeneity, and number” aimed to achieve a task through volunteer labor. We will return to this notion of volunteer-supplied labor and how participants are motivated later in this section.

There are several subtypes of crowdsourcing. The types we consider in this research study in the context of education are crowd creation, crowdvoting, crowdfunding, and crowd wisdom (Brabham 2008). Crowdfunding has an interesting potential application in education; its popular application thus far includes targets such profit-driven commercial debt distribution through to more altruistic projects such as disaster relief or ethics-driven arts support—e.g., label-less movie and music production. While crowdfunding may have a future role in research-led teaching and learning, our focus here will be on crowdvoting and crowd wisdom. This is because in the proposed system architecture, we intend to utilize these two features due to the knowledge created and being able to be exploited in the open learning settings. While not a new pedagogical concept, i.e., “hands up if,” or the anonymized modern equivalent of Clickers, crowdvoting techniques can effectively be applied to technology-enhanced learning. They can help collect and gauge a large crowd’s view on a certain area of learning, for example, if we are gauging satisfaction of learning materials or informally checking understanding of a learning outcome. Equally, crowd wisdom allows the aggregation of data in the form of problem solutions and sharing exercise workings between “crowds” of students. However, providing a technological solution is not necessarily in itself sufficient to produce these effects. Thus, beyond the desired collegiate effect of a cohort of students engaged together in studying, involving the “crowd” in a real participation is a key to the success of these technologies. There are some techniques, often identified to attract a crowd (Brabham 2008; Franklin et al. 2011). A popular technique is simply to financially reward crowd participants, awarding money or exchanging points for contributing to votes or giving information. A further technique concerns communities desiring crowd action that may provide entertainment to attract and retain crowds. Participants may receive a game, music, and film—as a reward for their contribution, or the entertainment may simply be used to attract them to the crowd.

There are already several existing crowdsourcing applications and communities used as online education support tools (Buecheler et al. 2010). An overview of examples, illustrative of the types of crowdsourced education, is shown in Table 1. They are then expanded on later in this section.

**Table 1:** Crowdsourcing application comparison

App	Overview	Crowd type	Challenges
Stack Overflow	Used to exchange questions and answers in topics of software development and programming.	Wisdom/voting	Unsuitable for novice programmers. Democratic-only assessment of quality.
Wikipedia	It is a wiki-based web application, which allows people to add, modify, or delete content in collaboration with others.	Wisdom	Scientific assessment to assess credibility of crowd inputs. They are only assessed “democratically.”
CourseEra	An educational and technological organization that offers free online courses in a wide range of topics to teach millions of students rather than hundreds.	Crowd creation	No long-term engagements for students. No collegiate support.

“Stack Overflow” is a crowdsourcing community application used in education to exchange questions and answers between programmers on a wide range of topics in computer programming. It can help professional programmers get a quick answer for their questions. However, it has a range of limitations as an educational tool. For example, it is not suitable for novice programmers as they need a unique level of detailed feedback (Chen et al. 2012; Aritajati and Narayanan 2013). More importantly, there is a distinct pattern describing who often contributes to the site and why there is a contribution. Some studies have reported most contributions in Stack Overflow come from constantly active contributors (Kim et al. 2013), whereas infrequent users post more queries than give answers. As such, this suggests that the motivation for those active and answer-providing contributors would be both altruism and the “reward” of non-currency reputation points. However, the altruism and community engagement help when active contributors earn more reputation points, and they gain site privileges, such as voting to delete answers.

“Wikipedia” is another example of a crowdsourcing platform. However, it can also be argued that Wikipedia is an online crowdsourced education repository. There is no formal application process to gain edit privileges on Wikipedia; in fact, most pages can be edited anonymously, safe for the user’s Internet Protocol (IP) address. Users who choose to register are not required to state or validate their experience or qualification background. Wikipedia does not have an assessment tool that assesses the inputs of the editors (crowd wisdom) to indicate whether their inputs are credible or not (Weld et al. 2012). Conversely, due to its massive user base, Wikipedia does have many advantages; it is a growing multilingual platform, and content is provided democratically, by consensus. While this in itself does not guarantee correct information—and could be potentially hostile to new research—there is a growing “citation needed” culture. This is where page editors may mark a document at a contentious or doubtful paragraph to request a citation to help readers verify whether the content is verifiable or not. Returning to the edit-by-consensus model, while this can stifle new work, it is still an important pedagogical aspect—peer review. The advantage of Wikipedia’s peer review model is that anyone with access to the Internet can edit the work of others or request a citation needed on information they do not think is common knowledge. Research has shown that peer review can improve the quality of published works by, for example, identifying scientific mistakes or wrong references (Weld et al. 2012). However, extreme care should still be employed when using Wikipedia as a reference material. There is a hierarchy whereby pages can be protected and edited only by authorized users. While the authorized user hierarchy is a loosely democratic structure, it is also corporatized—it receives criticism for being nepotistic, unequal, and inconsistent in its application of rules.

Massive open online course (MOOC) was proposed as a form of open education, with almost all of the teaching and learning occurring online. MOOC is designed to offer to the public an open access education, meaning it is open for anyone to sign up and learn whatever topic is being taught. “CourseEra” is a recent example of a massive open online course (Walker 2013). It is the product of an educational organization that offers free online courses in a wide range of topics to teach millions of students rather than hundreds. The CourseEra organization partners with top universities (crowd creation) in the world to offer online courses for free to anyone. It is still an immature market offer, and its revenue model is not yet clear; thus, its underlying ethos, while appearing altruistic, may still yet evolve or become marketized. Even at this early stage, several challenges have emerged. The first is the course retention rate; some students withdraw from the online courses at an early stage of the course. Weld et al. (2012) noted that under 15 % of students completed the Norvig/Thrun online Artificial Intelligence class and out of 104,000 students registering for Stanford’s 2011 Machine Learning class, 46,000 submitted the first assignment and 13,000 passed.

Crowdsourced applications, such as Stack Overflow, Wikipedia, and CourseEra, have emerged as a major problem-solving and data-gathering paradigm for online learning. Those applications offer a number of advantages to their users, for instance, getting a quick answer for programming-related posted question and giving a learner more freedom to participate and provide own opinions. However, they still have various drawbacks. For example, Stack Overflow was considered as an unsuitable application for novice programmers. This is because learning quality is poorly managed, and also, there is no differentiation between the expertise level of a professional and a novice programmer. Moreover, another challenge within this application refers to student engagements. Therefore, any proposed future technology-enhanced learning system must look to understand and address the issues behind poor retention (and the implied measure of engagement). For example, better engagement in an automated feedback e-learning system may be achieved by better differentiation of the learner’s ability. Previous work by the authors of this paper has explored learner differentiation by assessment (Alghamdi et al. 2013).

Finally, and closer to educational crowdsourcing, is the altruistic participation or community reward. Participants join the crowd for the reward of participating, the exchange of information. This is perhaps a more obvious draw when it comes to rewarding novice students—they will benefit from good quality information in the form of solution assistance and guided discussion. However, beyond the collegiate-like effect of gifted and talented students feeling personal satisfaction in helping less able students, it is not immediately obvious as to how these high-ability students can be attracted into participation. As such, investigation and experimentation must be conducted into the value of other reward systems for able students to increase their crowd participation. Tackling the issues identified in the previous sections and building on the findings of many studies that have been explored to gain insights on the recent limitations and obstacles of open learning solutions, the following section focuses on the architecture, structure, and functionalities of an emerging adaptive, student-centric system for open learning that considers the learner as an individual with his/her inherent abilities.

## Teaching, learning, and assessment for an intelligent, adaptive learning system in an open learning context

**Fig. 1:**
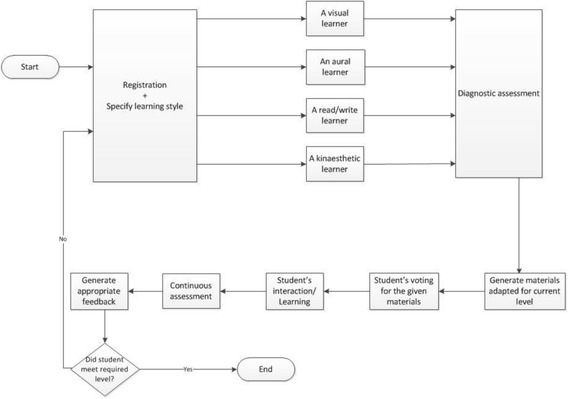
Learner-followed process in the proposed open learning adaptive system

The main inspiration for evolving current technology-enhanced learning approaches towards novel open learning system is the utilization of the advantages of crowdsourcing and adaptive systems. In addition, we consider the Assessment for Learning (AfL) initiative (Brown 2004), comprising diagnostic and continuous assessment as well as linking AfL with the preferred learning style of the learner. This defines a structured learning approach based on a student’s prior knowledge and student’s learning style preference, followed by learning informed by a student’s assessment performance. This methodology is applied in the proposed system, such that curriculum sequencing and material generation is fully integrated into an adaptive, student-centric learning system. This process is shown in Fig. 1.

When first-time learners enter this proposed adaptive system, they need to sign up to the system by using a registration form. The registration process ensures the privacy of the data, and all the entered information will be securely stored. Once a learner registers, a learner profile will be created to store all their information and will be saved in the student knowledge model. On the system, the VARK learning style model is employed, as it is one of the most influential and flexible models as explained previously in Section 2.1. When the registration is completed, the system will show the learner a short tutorial that explains the four styles in the VARK learning style model.

**Fig. 2:**
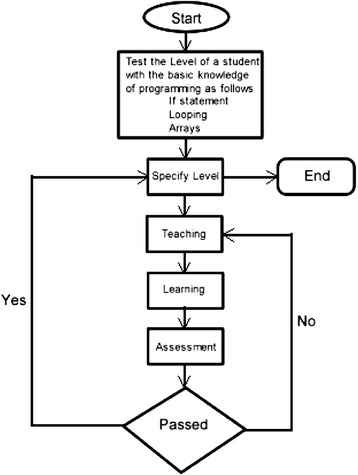
Data flowchart of the proposed open learning adaptive system

After this tutorial, the system proceeds with two significant diagnostic tasks with the learner. The first task is to determine the learner’s preferred style by asking him/her to fill in the online VARK questionnaire. This information is logged in the student’s knowledge model. The following task is to test the learner’s prior knowledge of the subject via diagnostic assessment, establishing the entry level of ability (see Fig. 2).

Upon completing this test, the system specifies the current level (Beginner-Intermediate-Advanced) of the student and directs him/her to the next step (Teaching). In this step, the system generates the appropriate curriculum material (suitable at entry level and transposed to the correct learning style) for the learner. Subsequently, the system takes the student to the “Learning” part of this phase, where he/she will deal with more programming examples with adequate intelligent help on each step of the problem-solving, including giving a hint to executing the next step and/or providing access to the crowdsourcing support. Following to that, the system takes the student to the “Assessment” part, as in this step, he/she will accomplish the most appropriate exam which can range from a simple question to a complex programming problem, whereas the system will provide code error feedback. Lastly, in case this student passes the assessment part, the system will specify a new level (until the student achieves the advanced programming level desired).

**Fig. 3:**
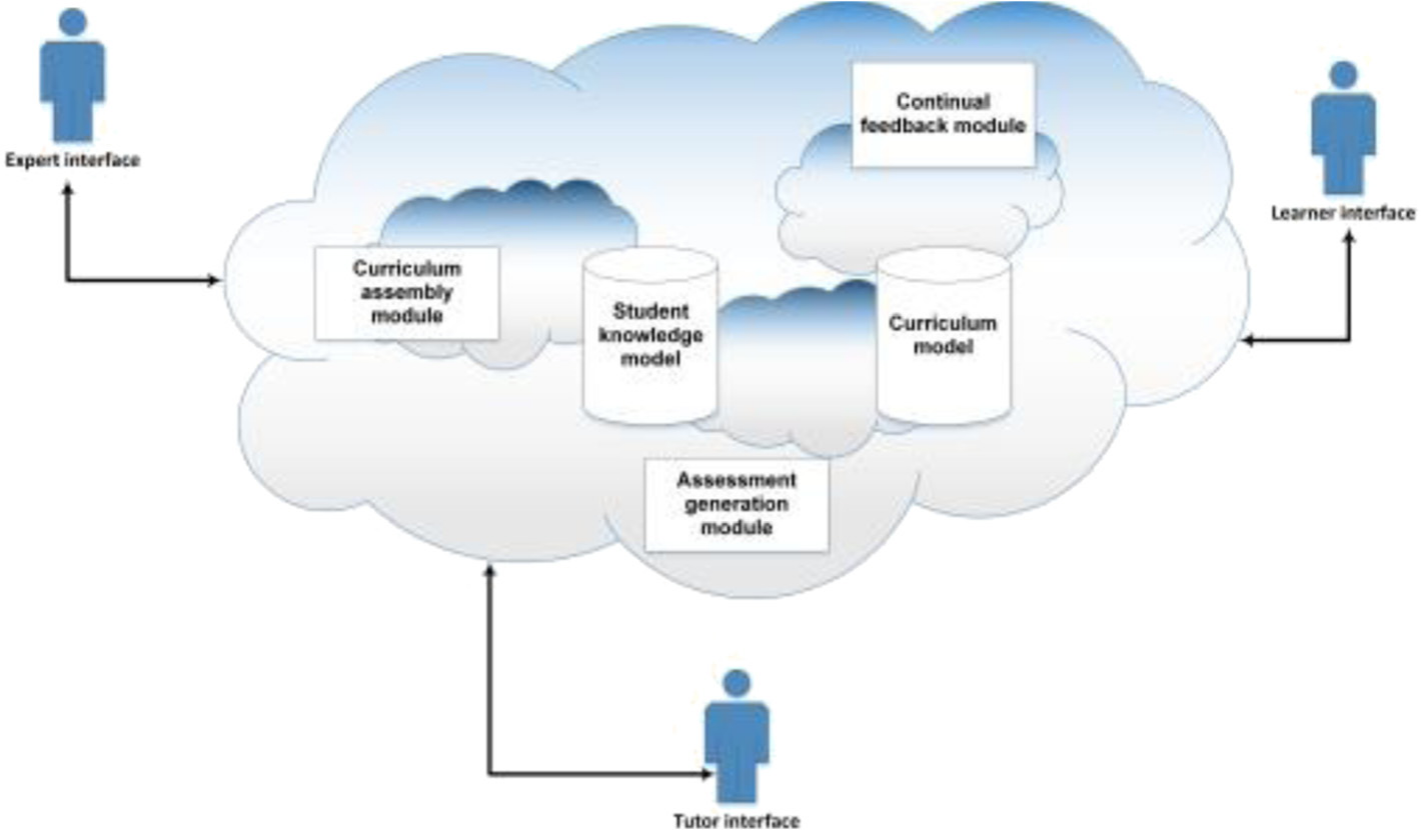
High-level system architecture showing example data models

The system will allow the learner to vote for the material that has been provided in order to accredit the level of satisfaction (crowdvoting). The student continues to be engaged in a formative “Continuous Assessment” that serves granting appropriate feedback and adapting the curriculum and learning styles appropriately. While the system framework architecture is a matter for the research work itself, it is envisaged at this early stage that the system will comprise a number of modules that use a variety of artificial intelligence techniques to interpret models defining features like the curriculum being followed (i.e., teaching materials, intended learning outcomes), a crowdsourcing model, for example, for distributed problem-solving and crowdvoting as well as the pedagogical aspects of this curriculum (e.g., appropriate assessment methods) and individual learner performance aspects (e.g., assessment results). The architecture of the proposed adaptive system is concerned with modeling and interpreting the data discussed over the previous sections to fulfill the pedagogical needs identified. A high-level overview of the architecture is shown in Fig. 3.

The system comprises two distinct knowledge models: the student knowledge/performance model and the curriculum model. These models are designed to maintain information on (1) current student knowledge, sourced from the assessment performance, and (2) curriculum knowledge, such as learning materials and outcomes, along with the assessment methods. Distinct modules communicate with one or both of these knowledge models, responsible for assembling and structuring learning, generating assessment materials, feedback, and regenerating curriculum materials. These modules are described in more detail in Table 2.

**Table 2:** Component/module overview

Component	Description
Student knowledge model	Contains information about individual student’s learning—ranging from materials studied, assessments taken, through to assessment results, extrapolated to identify performance against learning outcomes defined in the curriculum model.
Curriculum model	Stores curriculum-related data; at its lowest level, specifications of the learning outcomes that make up a unit with differentiation levels. This model is likely to maintain appropriate learning materials and assessment templates for relevant outcomes. This will be populated by tutors and experts to specify courses and modules.
Curriculum assembly	Adapts curriculum model data to produce a series of materials for a given set of learning outcomes, tailored to a specific student model.
Assessment generation	Transforms curriculum model data into appropriate assessments for either a set of learning outcomes provided either directly by a tutor or inferred from a student model’s outstanding learning outcomes.
Continual feedback	Produces feedback on assessment submissions, aligning student performance against learning outcomes in the curriculum models, using data from prior student attempts and ongoing tutor input.
Tutor, learner, and expert interface	Provides a user interface and access control for the various roles. The tutor and expert interfaces will provide intuitive mechanisms for inputting curriculum materials and manually checking assignments and student performance, while the learner interfaces will provide rich lecture, tutorial, and assignment user interfaces.

In order to achieve the methodological benefit of crowdsourced systems in open education, the pioneered system will incorporate three different platforms. The first one will be for novice programmers. The second platform will be allocated to those who have an intermediate level, whereas the last platform will be for learners who have advanced programming skills. The diagnostic assessment tool will be used to appoint a learner to a suitable platform. In this instance, every learner will be asked to take the diagnostic exam, and according to his/her performance, he/she will be assigned to the appropriate platform. For instance, platform number one will focus on the lower learning outcomes whereas the other two platforms will be designed for the intermediate and higher learning outcomes.

In all these planned platforms, learners will be given the opportunity to vote for the material, and also, learners who actively participate in their specified platforms will be visibly distinguished (for instance, higher privileges to add-delete) from those who do not participate very often. This will allow the learners to learn from the teaching material, on the one hand, and collaboratively gain experience from other learners (peer learners) as well as disseminate and share their experience, fully engaging in the crowdsourcing community, on the other hand. Furthermore, the proposed online adaptive system will consider the organization of student activities in those platforms. For example, students would be allowed to advise each other when they are solving a programming problem that would be weekly generated by the system; however, they will not be permitted to directly provide the answer as the aim of this work-in-progress online adaptive environment is to let all students think as a team and give everyone enough time in thinking how to solve a problem. At the end of the week, the system will post the model answer for the problem of interest. Additional challenges that will be considered include (1) investing how segregating on ability might adversely affect crowdsourcing, (2) the adverse effect that the proposed system have on student engagement, and (3) how the beneficial effect of reflective learning can be mapped onto the crowdsourced platforms for open learning.

## Summary and future work

Currently, education provision is encountering new challenges, radical inventions, and emerging technologies. The main area where innovation is presented addresses instructional methodologies that affect open communities. As such, modern education benefits from developments in intelligent systems and widespread high-speed Internet access. Adaptive, intelligent systems are increasingly gaining ground as a pedagogical delivery method yet still have far to go in terms of refining quality of materials, student performance, and engagement monitoring. Furthermore, there are challenges that stem from teaching increasingly complex, frequently multi-disciplinary, courses in a distributed manner, which raises the demand for appropriately designed intelligent systems that address various learners, in terms of distinctive available and/or desired skills and knowledge.

This paper has discussed significant challenges and existing issues around emerging crowdsourcing applications and intelligent adaptive systems that pioneer a new line of research and development in novel open education settings. It has also outlined the plan to tackle pedagogical concerns and simultaneously design individualized technology-enhanced learning solutions for software development students. Consequently, the proposed framework underpins the merge between an intelligent adaptive system and crowdsourcing approaches to be beneficially applied towards the improvement of teaching processes and programming skills, especially very useful for novice programmers, in an open learning context, combining the outcomes of traditional, and promising ground-breaking research to serve today’s needs.

Future work aims to evolve this system into a fully functional system that will be evaluated used first year undergraduate students on the “Introduction to Programming” module. Those students will be divided into two groups. The first group will be an experimental group (those who will be taught by using the proposed system), and the second group will be the control group (those who will be taught in the traditional way such as in a classroom). Three different comprehensive exams will then be used to evaluate the performance of those two groups and will be marked by the proposed system. The control group students will be given sufficient training time in the research system before undertaking the evaluation. However, this student group will have the option, if preferred, to undertake the evaluative exams in paper form, marked by a human teacher. Once those three exams are accomplished, a comparison between both groups’ achievements will be made to identify the learner’s level in each of those two groups. This evaluation will provide comparable results and clearly specify the learner’s performance in each group providing feedback for iterative system advancement.
